# Control Problems for Semilinear Neutral Differential Equations in Hilbert Spaces

**DOI:** 10.1155/2014/431978

**Published:** 2014-02-23

**Authors:** Jin-Mun Jeong, Seong Ho Cho

**Affiliations:** Department of Applied Mathematics, Pukyong National University, Busan 608-737, Republic of Korea

## Abstract

We construct some results on the regularity of solutions and the approximate controllability for neutral functional differential equations with unbounded principal operators in Hilbert spaces. In order to establish the controllability of the neutral equations, we first consider the existence and regularity of solutions of the neutral control system by using fractional power of operators and the local Lipschitz continuity of nonlinear term. Our purpose is to obtain the existence of solutions and the approximate controllability for neutral functional differential control systems without using many of the strong restrictions considered in the previous literature. Finally we give a simple example to which our main result can be applied.

## 1. Introduction

Let *H* and *V* be real Hilbert spaces such that *V* is a dense subspace in *H*. Let *U* be a Banach space of control variables. In this paper, we are concerned with the global existence of solution and the approximate controllability for the following abstract neutral functional differential system in a Hilbert space *H*:

(1) ddt[x(t)+(Bx)(t)]=Ax(t)+f(t,x(t))+(Cu)(t),t∈(0,T],x(0)=x0,  (Bx)(0)=y0,

where *A* is an operator associated with a sesquilinear form on *V* × *V* satisfying Gårding's inequality, *f* is a nonlinear mapping of [0,*T*] × *V* into *H* satisfying the local Lipschitz continuity, *B* : *L* ^2^(0,*T*;*V*) → *L* ^2^(0,*T*;*H*) and *C* : *L* ^2^(0,*T*;*U*) → *L* ^2^(0,*T*;*H*) are appropriate bounded linear mapping.

This kind of equations arises in population dynamics, in heat conduction in material with memory and in control systems with hereditary feedback control governed by an integrodifferential law.

Recently, the existence of solutions for mild solutions for neutral differential equations with state-dependence delay has been studied in the literature in [1, 2]. As for partial neutral integrodifferential equations, we refer to [3–6]. The controllability for neutral equations has been studied by many authors, for example, local controllability of neutral functional differential systems with unbounded delay in [7], neutral evolution integrodifferential systems with state dependent delay in [8, 9], impulsive neutral functional evolution integrodifferential systems with infinite delay in [10], and second order neutral impulsive integrodifferential systems in [11, 12]. Although there are few papers treating the regularity and controllability for the systems with local Lipschitz continuity, we can just find a recent article by Wang [13] in case of semilinear systems. Similar considerations of semilinear systems have been dealt with in many references [14–17].

In this paper, we propose a different approach from the earlier works (briefly introduced in [1–6] about the mild solutions of neutral differential equations. Our approach is that results of the linear cases of Di Blasio et al. [18] and semilinear cases of [19] on the *L* ^2^-regularity remain valid under the above formulation of the neutral differential equation (1). For the basics of our study, the existence of local solutions of (1) is established in *L* ^2^(0,*T*;*V*)∩*W* ^1,2^(0,*T*;V*)↪*C*([0,*T*];*H*) for some *T* > 0 by using fractional power of operators and Sadvoskii's fixed point theorem. Thereafter, by showing some variations of constant formula of solutions, we will obtain the global existence of solutions of (1) and the norm estimate of a solution of (1) on the solution space. Consequently, in view of the properties of the nonlinear term, we can take advantage of the fact that the solution mapping *u* ∈ *L* ^2^(0,*T*;*U*) ↦ *x* is Lipschitz continuous, which is applicable for control problems and the optimal control problem of systems governed by nonlinear properties.

The second purpose of this paper is to study the approximate controllability for the neutral equation (1) based on the regularity for (1); namely, the reachable set of trajectories is a dense subset of *H*. This kind of equations arises naturally in biology, physics, control engineering problem, and so forth.

The paper is organized as follows. In Section 2, we introduce some notations. In Section 3, the regularity results of general linear evolution equations besides fractional power of operators and some relations of operator spaces are stated. In Section 4, we will obtain the regularity for neutral functional differential equation (1) with nonlinear terms satisfying local Lipschitz continuity. The approach used here is similar to that developed in [13, 19] on the general semilinear evolution equations, which is an important role to extend the theory of practical nonlinear partial differential equations. Thereafter, we investigate the approximate controllability for the problem (1) in Section 5. Our purpose in this paper is to obtain the existence of solutions and the approximate controllability for neutral functional differential control systems without using many of the strong restrictions considered in the previous literature.

Finally, we give a simple example to which our main result can be applied.

## 2. Notations

Let *Ω* be a region in an *n*-dimensional Euclidean space ℝ^*n*^ and closure $\bar{\Omega }$ .

 
* C* ^*m*^(*Ω*) is the set of all *m*-times continuously differential functions on *Ω*.
 
* C*_0_^*m*^(*Ω*) will denote the subspace of *C* ^*m*^(*Ω*) consisting of these functions which have compact support in *Ω*.
 
* W* ^*m*,*p*^(*Ω*) is the set of all functions *f* = *f*(*x*) whose derivative *D* ^*α*^ *f* up to degree *m* in distribution sense belong to *L* ^*p*^(*Ω*). As usual, the norm is then given by


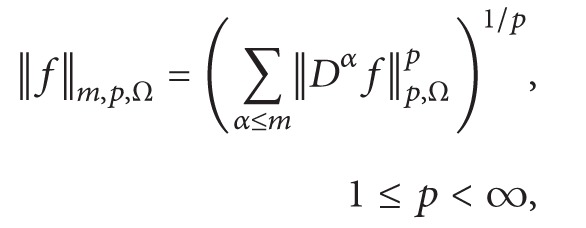

(2)



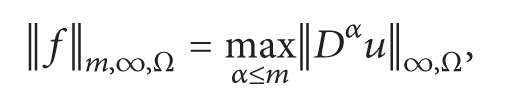

(3)

where *D* ^0^ *f* = *f*. In particular, *W* ^0,*p*^(*Ω*) = *L* ^*p*^(*Ω*) with the norm ||·||_*p*,*Ω*_.
 
* W*_0_^*m*,*p*^(*Ω*) is the closure of *C*_0_^*∞*^(*Ω*) in *W* ^*m*,*p*^(*Ω*).
 For *p* = 2 we denote *W* ^*m*,2^(*Ω*) = *H* ^*m*^(*Ω*) and *W*_0_^2,*p*^(*Ω*) = *H*_0_^*m*^(*Ω*).
 Let *p*′ = *p*/(*p* − 1),  1 < *p* < *∞*. *W* ^−1,*p*^(*Ω*) stands for the dual space *W*_0_^1,*p*′^(*Ω*)* of *W*_0_^1,*p*′^(*Ω*) whose norm is denoted by ||·||_−1,*p*,*∞*_.

If *X* is a Banach space and 1 < *p* < *∞*,

 
*L* ^*p*^(0,*T*;*X*) is the collection of all strongly measurable functions from (0,*T*) to *X*, the *p*th powers of norms are integrable,
 
*C* ^*m*^([0,*T*];*X*) will denote the set of all *m*-times continuously differentiable functions from [0,*T*] to *X*.
 If *X* and *Y* are two Banach spaces, *B*(*X*,*Y*) is the collection of all bounded linear operators from *X* to *Y*, and *B*(*X*,*X*) is simply written as *B*(*X*).
 For an interpolation couple of Banach spaces *X*_0_ and *X*_1_, (*X*_0_, X_1_)_*θ*,*p*_ and [*X*_0_,*X*_1_]_*θ*_ denote the real and complex interpolation spaces between *X*_0_ and *X*_1_, respectively.

Let *A* be a closed linear operator in a Banach space. Then

 
*D*(*A*) denotes the domain of (*A*) and *R*(*A*) the range of *A*;
 
*ρ*(*A*) denotes the resolvent set of *A*, *σ*(*A*) the spectrum of *A*, and *σ*_*p*_(*A*) the point spectrum of *A*;
 the kernel or null space {*x* ∈ *D*(*A*) : *Ax* = 0} of *A* is denoted by Ker⁡(*A*).

## 3. Regularity for Linear Equations

If *H* is identified with its dual space we may write *V* ⊂ *H* ⊂ V* densely and the corresponding injections are continuous. The norm on *V*, *H*, and V* will be denoted by ||·||, |·| and ||·||_∗_, respectively. The duality pairing between the element *v*_1_ of V* and the element *v*_2_ of *V* is denoted by (*v*_1_,*v*_2_), which is the ordinary inner product in *H* if *v*_1_, *v*_2_ ∈ *H*.

For *l* ∈ V* we denote (*l*,*v*) by the value *l*(*v*) of *l* at *v* ∈ *V*. The norm of *l* as element of V* is given by

(4) $$\begin{array}{c} {||l||}_{*}=\underset{v\in V}{\sup}\frac{\left|l,v\right|}{||v||}. \end{array}$$

Therefore, we assume that *V* has a stronger topology than *H* and, for brevity, we may consider

(5) $$\begin{array}{c} {||u||}_{*}\leq \left|u\right|\leq ||u||,\;\forall u\in V. \end{array}$$

Let *a*(·,·) be a bounded sesquilinear form defined in *V* × *V* and satisfying Gårding's inequality:

(6) Rea(u,u)≥δ||u||2, δ>0.

Let *A* be the operator associated with this sesquilinear form:

(7) $$\begin{array}{c} \left(Au,v\right)=a\left(u,v\right),\;u,v\in V. \end{array}$$

Then *A* is a bounded linear operator from *V* to V* by the Lax-Milgram theorem. The realization of *A* in *H* which is the restriction of *A* to

(8) $$\begin{array}{c} D\left(A\right)=\left\{u\in V\;:\;Au\in H\right\} \end{array}$$

is also denoted by *A*. From the following inequalities

(9) δ||u||2≤Rea(u,u)≤C|Au||u|≤C||u||D(A)|u|,

where

(10) $$\begin{array}{c} {||u||}_{D(A)}={\left({\left|Au\right|}^{2}+{\left|u\right|}^{2}\right)}^{1/2} \end{array}$$

is the graph norm of *D*(*A*), it follows that there exists a constant *C*_0_ > 0 such that

(11) $$\begin{array}{c} ||u||\leq C_{0}{||u||}_{D\left(A\right)}^{1/2}{\left|u\right|}^{1/2}. \end{array}$$

Thus we have the following sequence:

(12) $$\begin{array}{c} D\left(A\right)\subset V\subset H\subset V^{*}\subset D{\left(A\right)}^{*}, \end{array}$$

where each space is dense in the next one and continuous injection.

**Lemma 1**

With the notations (11), (12), one has

(13) $$\begin{array}{c} {\left(V,V^{*}\right)}_{1/2,2}=H,\\ {\left(D\left(A\right),H\right)}_{1/2,2}=V, \end{array}$$

where (*V*,V*)_1/2,2_ denotes the real interpolation space between *V* and V*(Section 1.3.3 of [20]).

It is also well known that *A* generates an analytic semigroup *S*(*t*) in both *H* and V*. The following lemma is from Lemma 3.6.2 of [21].

**Lemma 2**

Let *S*(*t*) be the semigroup generated by −*A*. Then there exists a constant *M* such that

(14) $$\begin{array}{c} \left|S\left(t\right)\right|\leq M,\;\;{||s\left(t\right)||}_{*}\leq M. \end{array}$$

For all *t* > 0 and every *x* ∈ *H* or V* there exists a constant *M* > 0 such that the following inequalities hold:

(15) $$\begin{array}{c} \left|S\left(t\right)x\right|\leq {Mt}^{-1/2}{||x||}_{*},\;\;||S\left(t\right)x||\leq {Mt}^{-1/2}\left|x\right|. \end{array}$$

By virtue of (6), we have that 0 ∈ *ρ*(*A*) and the closed half plane {*λ* : *Reλ* ≥ 0} is contained in the resolvent set of *A*. In this case, there exists a neighborhood *U* of 0 such that

(16) $$\begin{array}{c} \rho \left(A\right)⊃\left\{\lambda :\left|\arg\lambda \right|>\omega \right\}\cup U. \end{array}$$

Hence, we can choose that the path Γ runs in the resolvent set of *A* from *∞e* ^*iθ*^ to *∞e* ^−*iθ*^, *ω* < *θ* < *π*, avoiding the negative axis. For each *α* > 0, we put

(17) $$\begin{array}{c} A^{-\alpha }=\frac{1}{2\pi i}\overset{}{\underset{\Gamma }{\int }}\lambda^{-\alpha }{\left(A-\lambda \right)}^{-1}d\lambda , \end{array}$$

where *λ* ^−*α*^ is chosen to be for *λ* > 0. By assumption, *A* ^−*α*^ is a bounded operator. So we can assume that there is a constant *M*_0_ > 0 such that

(18) $$\begin{array}{c} {||A^{-\alpha }||}_{ℒ(H)}\leq M_{0},\;\;{||A^{-\alpha }||}_{ℒ(V^{*},V)}\leq M_{0}. \end{array}$$

For each *α* ≥ 0, we define an operator *A* ^*α*^ as follows:

(19) $$\begin{array}{c} A^{\alpha }=\{\begin{array}{cc} {\left(A^{-\alpha }\right)}^{-1} & \text{for}\;\alpha >0,\\ I & \text{for}\;\alpha =0. \end{array} \end{array}$$

The subspace *D*(*A* ^*α*^) is dense in *H* and the expression

(20) $$\begin{array}{c} {||x||}_{\alpha }=||A^{\alpha }x||,\;x\in D\left(A^{\alpha }\right) \end{array}$$

defines a norm on *D*(*A* ^*α*^).

**Lemma 3**

(a)  *A* ^*α*^ is a closed operator with its domain dense.

(b) If 0 < *α* < *β*, then *D*(*A* ^*α*^)⊃*D*(*A* ^*β*^).

(c) For any *T* > 0, there exists a positive constant *C*_*α*_ such that the following inequalities hold for all *t* > 0:

(21) $$\begin{array}{c} {||A^{\alpha }S\left(t\right)||}_{ℒ(H)}\leq \frac{C_{\alpha }}{t^{\alpha }},\;\;{||A^{\alpha }S\left(t\right)||}_{ℒ(H,V)}\leq \frac{C_{\alpha }}{t^{3\alpha /2}}. \end{array}$$

**Proof**

From [21, Lemma 3.6.2] it follows that there exists a positive constant *C* such that the following inequalities hold for all *t* > 0 and every *x* ∈ *H* or V*:

(22) $$\begin{array}{c} \left|AS\left(t\right)x\right|\leq \frac{C}{t}\left|x\right|,\;\;||AS\left(t\right)x||\leq \frac{C}{t^{3/2}}\left|x\right|, \end{array}$$

which implies (21) by properties of fractional power of *A*. For more details about the above lemma, we refer to [21, 22].

Let the solution spaces *𝒲*(*T*) and *𝒲*_1_(*T*) of strong solutions be defined by

(23) $$\begin{array}{c} 𝒲\left(T\right)=L^{2}\left(0,T;D\left(A\right)\right)\cap W^{1,2}\left(0,T;H\right),\\ {𝒲}_{1}\left(T\right)=L^{2}\left(0,T;V\right)\cap W^{1,2}\left(0,T;V^{*}\right). \end{array}$$

Here, we note that by using interpolation theory, we have

(24) $$\begin{array}{c} 𝒲\left(T\right)\subset C\left(\left[0,T\right];V\right),\;\;{𝒲}_{1}\left(T\right)\subset C\left(\left[0,T\right];H\right). \end{array}$$

Thus, there exists a constant *M*_1_ > 0 such that

(25) $$\begin{array}{c} {||x||}_{C([0,T];V)}\leq M_{1}{||x||}_{𝒲(T)},\;\;{||x||}_{C([0,T];H)}\leq M_{1}{||x||}_{{𝒲}_{1}(T)}. \end{array}$$

First of all, consider the following linear system:

(26) $$\begin{array}{c} x^{\prime }\left(t\right)+Ax\left(t\right)=k\left(t\right),\\ x\left(0\right)=x_{0}. \end{array}$$

By virtue of Theorem 3.3 of [6] (or Theorem 3.1 of [3, 21]), we have the following result on the corresponding linear equation of (26).

**Lemma 4**

Suppose that the assumptions for the principal operator *A* stated above are satisfied. Then the following properties hold:

(1) for *x*_0_ ∈ *V* = (*D*(*A*), *H*)_1/2,2_  (see Lemma 1) and *k* ∈ *L* ^2^(0,*T*;*H*), *T* > 0, there exists a unique solution *x* of (26) belonging to *𝒲*(*T*) ⊂ *C*([0,*T*];*V*) and satisfying
(27) $$\begin{array}{c} {||x||}_{𝒲(T)}\leq C_{1}\left(||x_{0}||+{||k||}_{L^{2}(0,T;H)}\right), \end{array}$$
  where *C*_1_ is a constant depending on *T*;
(2) let *x*_0_ ∈ *H* and *k* ∈ *L* ^2^(0,*T*;V*), *T* > 0; then there exists a unique solution *x* of (26) belonging to *𝒲*_1_(*T*) ⊂ *C*([0,*T*];*H*) and satisfying
(28) $$\begin{array}{c} {||x||}_{{𝒲}_{1}(T)}\leq C_{1}\left(\left|x_{0}\right|+{||k||}_{L^{2}(0,T;V^{*})}\right), \end{array}$$
  where *C*_1_ is a constant depending on *T*.

**Lemma 5**

For every *k* ∈ *L* ^2^(0,*T*;*H*), let *x*(*t*) = ∫_0_^*t*^ *S*(*t* − *s*)*k*(*s*)*ds* for 0 ≤ *t* ≤ *T*. Then there exists a constant *C*_2_ such that

(29) $$\begin{array}{c} {||x||}_{L^{2}(0,T;V)}\leq C_{2}\sqrt{T}{||k||}_{L^{2}(0,T;H)}. \end{array}$$

**Proof**

By (27) we have

(30) $$\begin{array}{c} {||x||}_{L^{2}(0,T;D(A))}\leq C_{1}{||k||}_{L^{2}(0,T;H)}. \end{array}$$

Since

(31) ||x||L2(0,T;H)2=∫0T|∫0tS(t−s)k(s)ds|2dt≤M∫0T(∫0t|k(s)|ds)2dt≤M∫0Tt∫0t|k(s)|2dsdt≤MT22∫0T|k(s)|2ds,

it follows that

(32) $$\begin{array}{c} {||x||}_{L^{2}(0,T;H)}\leq T\sqrt{M/2}{||k||}_{L^{2}(0,T;H)}. \end{array}$$

From (11), (30), and (32) it holds that

(33) $$\begin{array}{c} {||x||}_{L^{2}(0,T;V)}\leq C_{0}\sqrt{C_{1}T}{\left(\frac{M}{2}\right)}^{1/4}{||k||}_{L^{2}(0,T;H)}. \end{array}$$

So, the proof is completed.

## 4. Semilinear Differential Equations

Consider the following abstract neutral functional differential system:

(34) ddt[x(t)+(Bx)(t)]=Ax(t)+f(t,x(t))+k(t),                 t∈(0,T],x(0)=x0,  (Bx)(0)=y0.

Then we will show that the initial value problem (34) has a solution by solving the integral equation:

(35) x(t)=S(t)[x0+y0]−(Bx)(t) +∫0tAS(t−s)Bx(s)ds +∫0tS(t−s){f(s,x(s))+k(s)}ds.

Now we give the basic assumptions on the system (34).

**Assumption B**

Let *B* : *L* ^2^(0,*T*;*V*) → *L* ^2^(0,*T*;*H*) be a bounded linear mapping such that there exist constants *β* > 1/3, *L* > 0, and a continuous nondecreasing function *b*(*t*):[0,*T*] → ℝ with *b*(0) = 0 such that

(36) $$\begin{array}{c} {||A^{\beta }Bx||}_{L^{2}(0,t;H)}\leq b\left(t\right){||x||}_{L^{2}(0,t;V)},\\ \forall \left(t,x\right)\in \left(0,T\right]\times L^{2}\left(0,T;V\right). \end{array}$$

**Assumption F**

*f* is a nonlinear mapping of [0, *T*] × *V* into *H* satisfying the following.

(i) There exists a function *L*_1_ : ℝ_+_ → ℝ such that
(37) $$\begin{array}{c} \left|f\left(t,x\right)-f\left(t,y\right)\right|\leq L_{1}\left(r\right)||x-y||,\;t\in \left[0,T\right], \end{array}$$
  hold for ||*x*||≤*r* and ||*y*||≤*r*.
(ii) The inequality
(38) $$\begin{array}{c} \left|f\left(t,x\right)\right|\leq L_{1}\left(r\right)\left(||x||+1\right) \end{array}$$
  holds for every *t* ∈ [0,*T*] and *x* ∈ *V*.

Let us rewrite (*Fx*)(*t*) = *f*(*t*,*x*(*t*)) for each *x* ∈ *L* ^2^(0,*T*;*V*). Then there is a constant, denoted again by *L*_1_(*r*), such that

(39) $$\begin{array}{c} {||Fx||}_{L^{2}(0,T;H)}\leq L_{1}\left(r\right)\left({||x||}_{L^{2}(0,T;V)}+1\right),\\ {||Fx_{1}-Fx_{2}||}_{L^{2}(0,T;H)}\leq L_{1}\left(r\right){||x_{1}-x_{2}||}_{L^{2}(0,T;V)} \end{array}$$

hold for *x* ∈ *L* ^2^(0,*T*;*V*) and *x*_1_,*x*_2_ ∈ *B*_*r*_(*T*) = {*x* ∈ *L* ^2^(0,*T*;*V*):||*x*||_*L* ^2^(0,*T*;*V*)_ ≤ *r*}.

From now on, we establish the following results on the solvability of (34).

**Theorem 6**

Let Assumptions B and F be satisfied. Assume that *x*_0_ ∈ *H*, *k* ∈ *L* ^2^(0,*T*;V*) for *T* > 0. Then, there exists a solution *x* of (34) such that

(40) $$\begin{array}{c} x\in {𝒲}_{1}\left(T\right)\equiv L^{2}\left(0,T;V\right)\cap W^{1,2}\left(0,T;V^{*}\right)\subset C\left(\left[0,T\right];H\right). \end{array}$$

Moreover, there is a constant *C*_3_ independent of *x*_0_ and the forcing term *k* such that

(41) $$\begin{array}{c} {||x||}_{{𝒲}_{1}(T)}\leq C_{3}\left(1+\left|x_{0}\right|+{||k||}_{L^{2}(0,T;V^{*})}\right). \end{array}$$

One of the main useful tools in the proof of existence theorems for functional equations is the following Sadvoskii's fixed point theorem.

**Lemma 7 (see [23])**

Suppose that Σ is a closed convex subset of a Banach space *X*. Assume that *K*_1_ and *K*_2_ are mappings from Σ to *X* such that the following conditions are satisfied:

- (*K*_1_ + *K*_2_)(Σ) ⊂ Σ,
- *K*_1_ *is a completely continuous mapping*,
- *K*_2_ *is a contraction mapping*.

Then the operator *K*_1_ + *K*_2_ has a fixed point in Σ.

**Proof of Theorem 6**

Let

(42) $$\begin{array}{c} r_{0}=2C_{1}\left|x_{0}+y_{0}\right|, \end{array}$$

where *C*_1_ is constant in Lemma 4. Let *β* > 1/3, and choose 0 < *T*_1_ < *T* such that

(43) T13β/2[{C2L1(r0)(r0+1)+C2||k||L2(0,T1;V)}   + 2r0b(T1)C1−β(3β)−1/2(3β−2)−1] +r0M0b(T1)≤C1|x0+y0|,

where *C*_2_ is constant in Lemma 5. Let

(44) M^≡T13β/2{C2L1(r0)+2(3β)−1/2(3β−2)−1C1−βb(T1)} +M0b(T1)<1.

Define a mapping *J* : *L* ^2^(0,*T*_1_;*V*) → *L* ^2^(0,*T*_1_;*V*) as

(45) (Jx)(t)=S(t)(x0+y0)−(Bx)(t) +∫0tAS(t−s)(Bx)(s)ds +∫0tS(t−s){f(s,x(s))+k(s)}ds.

It will be shown that the operator *J* has a fixed point in the space *L* ^2^(0,*T*_1_;*V*). By Assumptions B and F, it is easily seen that *J* is continuous from *C*([0,*T*_1_];*H*) in itself. Let

(46) $$\begin{array}{c} \Sigma =\left\{x\in L^{2}\left(0,T_{1};V\right):{||x||}_{L^{2}(0,T_{1};V)}\leq r_{0},\;\;x\left(0\right)=x_{0}\right\}, \end{array}$$

which is a bounded closed subset of *L* ^2^(0,*T*_1_;*V*). From (27) it follows that

(47) $$\begin{array}{c} {||S\left(\cdot \right)\left(x_{0}+y_{0}\right)||}_{L^{2}(0,T_{1};V)}\leq C_{1}\left|x_{0}+y_{0}\right|. \end{array}$$

By (21), (25), and assumption B we have

(48) ||Bx||L2(0,T1;V)=||A−βAβBx||L2(0,T1;V)≤||A−β||ℒ(H,V)||AβBx||L2(0,T1;H)≤r0M0b(T1).

By virtue of (29) in Lemma 5, for 0 < *t* < *T*_1_, it holds that

(49) ||∫0tS(t−s){f(s,x(s))+k(s)}ds||L2(0,T1;V) ≤C2T1||Fx+k||L2(0,T1;H) ≤C2T1{L1(r0)(||x||L2(0,T1;V)+1)+||k||L2(0,T1;V)} ≤C2T1{L1(r0)(r0+1)+||k||L2(0,T1;V)}.

Since (21) and Assumption F the following inequality holds:

(50) ||AS(t−s)Bx(s)||=||A1−βS(t−s)AβBx(s)||≤C1−β(t−s)3(1−β)/2r0b(T1).

Let

(51) $$\begin{array}{c} \left(Wx\right)\left(t\right)=\overset{t}{\underset{0}{\int }}AS\left(t-s\right)Bx\left(s\right)ds. \end{array}$$

Then there holds

(52) ||Wx||L2(0,T1;V)=[∫0T1||∫0tAS(t−s)Bx(s)ds||2dt]1/2≤[∫0T1(∫0tC1−β(t−s)3(1−β)/2r0b(T1)ds)2dt]1/2≤2r0b(T1)C1−β(3β−2)−1(∫0T1t3β−1dt)1/2=2r0b(T1)C1−β(3β)−1/2(3β−2)−1T13β/2.

Therefore, from (43), (47)–(52) it follows that

(53) ||Jx||L2(0,T1;V) ≤C1|x0+y0|+r0M0b(T1)  +T13β/2[{C2L1(r0)(r0+1)+C2||k||L2(0,T1;V)}     + 2(3β)−1/2(3β−2)−1r0b(T1)C1−β]≤r0,

and hence *J* maps Σ into Σ.

Define mapping *K*_1_ + *K*_2_ on *L* ^2^(0,*T*_1_;*V*) by the formula

(54) (Jx)(t)=(K1x)(t)+(K2x)(t),(K1x)(t)=−(Bx)(t),(K2x)(t)=S(t)(x0+y0) +∫0tAS(t−s)(Bx)(s)ds +∫0tS(t−s){f(s,x(s))+k(s)}ds.

We can now employ Lemma 7 with Σ. Assume that a sequence {*x*_*n*_} of *L* ^2^(0,*T*_1_;*V*) converges weakly to an element *x*_*∞*_ ∈ *L* ^2^(0,*T*_1_;*V*); that is, *w* − lim⁡_*n*→*∞*_ *x*_*n*_ = *x*_*∞*_. Then we will show that

(55) $$\begin{array}{c} \underset{n\to \infty }{\lim}||K_{1}x_{n}-K_{1}x_{\infty }||=0, \end{array}$$

which is equivalent to the completely continuity of *K*_1_ since *L* ^2^(0,*T*_1_;*V*) is reflexive. For a fixed *t* ∈ [0,*T*_1_], let *x*_t_*(*x*) = (*K*_1_ *x*)(*t*) for every *x* ∈ *L* ^2^(0,*T*_1_;*V*). Then *x*_t_* ∈ *L* ^2^(0,*T*_1_;V*) and we have lim⁡_*n*→*∞*_ *x*_t_*(*x*_*n*_) = *x*_t_*(*x*_*∞*_) since *w* − lim⁡_*n*→*∞*_ *x*_*n*_ = *x*_*∞*_. Hence,

(56) $$\begin{array}{c} \underset{n\to \infty }{\lim}\left(K_{1}x_{n}\right)\left(t\right)=\left(K_{1}x_{\infty }\right)\left(t\right),\;t\in \left[0,T_{1}\right]. \end{array}$$

By (21), (25), and assumption B we have

(57) ||(K1x)(t)||=||(Bx)(t)||=||A−βAβBx(t)||≤||A−β||ℒ(H,V)||AβBx||L2(0,T1;H)≤∞.

Therefore, by Lebesgue's dominated convergence theorem it holds that

(58) $$\begin{array}{c} \underset{n\to \infty }{\lim}\overset{T_{1}}{\underset{0}{\int }}{||\left(K_{1}x_{n}\right)\left(t\right)||}^{2}dt=\overset{T_{1}}{\underset{0}{\int }}{||\left(K_{1}x_{\infty }\right)\left(t\right)||}^{2}dt; \end{array}$$

that is, lim⁡_*n*→*∞*_||*K*_1_ *x*_*n*_||_*L* ^2^(0,*T*_1_;*V*)_ = ||*K*_1_ *x*_*∞*_||_*L* ^2^(0,*T*_1_;*V*)_. Since *L* ^2^(0,*T*_1_;*V*) is a Hilbert space, the relation (55) holds. Next, we prove that *K*_2_ is a contraction mapping on Σ. Indeed, for every *x*_1_ and *x*_2_ ∈ Σ, we have

(59) (K2x1)(t)−(K2x2)(t) =∫0tAS(t−s){(Bx1)(s)−(Bx2)(t)}ds  +∫0tS(t−s){f(s,x1(s))−f(s,x2(s))}ds.

Similar to (49) and (52), we have

(60) ||K2x1−K2x2||L2(0,T1;V) ≤T13β/2{C2L1(r0)+2(3β)−1/2     × (3β−2)−1C1−βb(T1)}  ×||x1−x2||L2(0,T1;V).

So by virtue of condition (44) the contraction mapping principle gives that the solution of (34) exists uniquely in [0,*T*_1_].

So by virtue of condition (44), *K*_2_ is contractive. Thus, Lemma 7 gives that the equation of (34) has a solution in *𝒲*_1_(*T*_1_).

From now on we establish a variation of constant formula (41) of solution of (34). Let *x* be a solution of (34) and *x*_0_ ∈ *H*. Then we have that from (47)-(52) it follows that

(61) ||x||L2(0,T1;V) ≤C1|x0+y0|+M0b(T1)||x||L2(0,T1;V)  +T13β/2[{C2L1(r0)(||x||L2(0,T1;V∗)+1)      + C2||k||L2(0,T1;V∗)}+2(3β)−1/2      ×(3β−2)−1C1−βb(T1)||x||L2(0,T1;V)].

Taking into account (44) there exists a constant *C*_3_ such that

(62) ||x||L2(0,T1;V) ≤(1−M^)−1  ×[C1|x0+y0|+r0M0b(T1)+T13β/2    ×{C2L1(r0)+C2||k||L2(0,T1;V∗)}] ≤C3(1+|x0|+||k||L2(0,T1;V∗))

which obtain the inequality (41). Since the conditions (43) and (44) are independent of initial value and by (25)

(63) $$\begin{array}{c} \left|x\left(T_{1}\right)\right|\leq {||x||}_{C(\left[0,T_{1};H\right])}\leq M_{1}{||x||}_{{𝒲}_{1}(T)}, \end{array}$$

by repeating the above process, the solution can be extended to the interval [0,*T*].

**Corollary 8**

If *M*_0_ *b*(*T*_1_) < 1, then the uniqueness of the solution of (34) in *𝒲*_1_(*T*) is obtained.

**Proof**

Let *M*_0_ *L* < 1. Then instead of condition (44), we can choose *T*_1_ such that

(64) M0b(T1)+T13β/2{C2L1(r0)+2(3β)−1/2        ×(3β−2)−1C1−βb(T1)}<1.

For every *x*_1_ and *x*_2_ ∈ Σ, we have

(65) (Jx1)(t)−(Jx2)(t) =(Bx2)(t)−(Bx1)(t)  +∫0tAS(t−s){Bx1(s)−Bx2(t)}ds  +∫0tS(t−s){f(s,x1(s))−f(s,x2(s))}ds.

Similar to (49) and (52), we have

(66) ||Jx1−Jx2||L2(0,T1;V) ≤[M0b(T1)+T13β/2{C2L1(r0)+2(3β)−1/2(3β−2)−1          × C1−βb(T1)}]||x1−x2||L2(0,T1;V).

So by virtue of condition (64) the contraction mapping principle gives that the solution of (34) exists uniquely in [0, *T*_1_].

**Remark 9**

Let Assumptions B and F be satisfied and (*x*_0_,*k*) ∈ *D*(*A*) × *L* ^2^(0,*T*;*H*). Then by the argument of the proof of Theorem 6 term by term, we also obtain that there exists a solution *x* of (34) such that

(67) x∈𝒲(T) ≡L2(0,T;D(A))∩W1,2(0,T;H)⊂C([0,T];V).

Moreover, there exists a constant *C*_3_ such that

(68) $$\begin{array}{c} {||x||}_{𝒲(T)}\leq C_{3}\left(1+||x_{0}||+{||k||}_{L^{2}(0,T;H)}\right), \end{array}$$

where *C*_3_ is a constant depending on *T*.

The following inequality is refered to as the Young inequality.

**Lemma 10 (Young inequality)**

Let *a* > 0,  *b* > 0, and 1/*p* + 1/*q* = 1, where 1 ≤ *p* < *∞*, and 1 < *q* < *∞*. Then for every *λ* > 0 one has

(69) $$\begin{array}{c} ab\leq \frac{\lambda^{p}a^{p}}{p}+\frac{b^{q}}{\lambda^{q}q}. \end{array}$$

From the following result, we obtain that the solution mapping is continuous, which is useful for physical applications of the given equation.

**Theorem 11**

Let Assumptions B and F be satisfied and (*x*_0_,*y*_0_,*k*) ∈ *H* × *H* × *L* ^2^(0,*T*;V*). Then the solution *x* of (34) belongs to *x* ∈ *𝒲*_1_(*T*) ≡ *L* ^2^(0,*T*;*V*)∩*W* ^1,2^(0,*T*;V*) and the mapping

(70) $$\begin{array}{c} H\times H\times L^{2}\left(0,T;V^{*}\right)∋\left(x_{0},y_{0},k\right)⟼x\in {𝒲}_{1}\left(T\right) \end{array}$$

is continuous.

**Proof**

From Theorem 6, it follows that if (*x*_0_,*k*) ∈ *H* × *L* ^2^(0,*T*;V*), then *x* belongs to *𝒲*_1_(*T*). Let (*x*_0*i*_,*y*_0*i*_,*k*_*i*_) ∈ *H* × *H* × *L* ^2^(0,*T*;V*) and let *x*_*i*_ ∈ *𝒲*_1_(*T*) be the solution of (34) with (*x*_0*i*_,*y*_0*i*_,*k*_*i*_) in place of (*x*_0_,*y*_0_,*k*) for *i* = 1,2. Let *x*_*i*_  (*i* = 1,2) ∈ Σ. Then as seen in Theorem 6, it holds that

(71) ddt[x1(t)−x2(t)+(Bx1)(t)−(Bx2)(t)] =A(x1(t)−x2(t))+f(t,x1(t))−f(t,x2(t))  +k1(t)−k2(t),

(72) $$\begin{array}{c} x_{1}\left(0\right)-x_{2}\left(0\right)=x_{01}-x_{02}. \end{array}$$

So the solution of the above equation is represented by

(73) x1(t)−x2(t) =S(t){(x01−x02)+(y01−y02)}  +(Bx2)(t)−(Bx1)(t)  +∫0tAS(t−s){(Bx1)(t)−(Bx2)(t)}ds  +∫0tS(t−s){f(s,x1(t))        −f(s,x2(s)+k1(s)−k2(s)}ds.

And, hence, it holds that

(74) ||x1−x2||L2(0,T1;V) ≤C1(|x01−x02|+|y01−y02|)  +C2T13β/2||k1−k2||L2(0,T1;V∗)  +T13β/2{M0L+C2L1(r)+2(3β)−1/2      × (3β−2)−1b(T1)C1−β}  ×||x1−x2||L2(0,T1;V).

From (43), we have

(75) ||x1−x2||L2(0,T1;V) ≤(1−M^)−1(C1(|x01−x02|+|y01−y02|)        + C2T13β/2||k1−k2||L2(0,T1;V∗)).

Hence, repeating this process as seen in Theorem 6, we conclude that the solution mapping is continuous.

For *k* ∈ *L* ^2^(0,*T*;V*), let *x*_*k*_ be the solution of (34) with *k* instead of *Bu*.

**Theorem 12**

Let one assume that the embedding *V* ⊂ *H* is compact. For *k* ∈ *L* ^2^(0,*T*;V*) let *x*_*k*_ be the solution of (34). Then the mapping *k* ↦ *x*_*k*_ is compact from *L* ^2^(0,*T*;V*) to *L* ^2^(0,*T*;*H*). Moreover, if one defines the operator *ℱ* by

(76) $$\begin{array}{c} ℱ\left(k\right)=f\left(\cdot ,x_{k}\right), \end{array}$$

then *ℱ* is also a compact mapping from *L* ^2^(0,*T*;V*) to *L* ^2^(0,*T*;*H*).

**Proof**

If (*x*_0_,*k*) ∈ *H* × *L* ^2^(0,*T*;V*), then in view of Theorem 6

(77) $$\begin{array}{c} {||y_{k}||}_{{𝒲}_{1}(T)}\leq C_{2}\left(\left|x_{0}\right|+{||k||}_{L^{2}(0,T;V^{*})}\right). \end{array}$$

Since *x*_*k*_ ∈ *L* ^2^(0,*T*;*V*), we have *f*(·,*x*_*k*_) ∈ *L* ^2^(0,*T*;*H*). Consequently, by (25), we know that *x*_*k*_ ∈ *𝒲*_1_(*T*) ⊂ *C*([0,*T*];*H*). With aid of (*a*) of Lemma 3, noting that ||*x*_*k*_||_*L* ^2^(0,*T*;*V*)_ ≤ ||*x*_*k*_||_*𝒲*_1_(*T*)_, we have

(78) $$\begin{array}{c} {||x_{k}||}_{{𝒲}_{1}(T)}\leq C_{3}\left(1+\left|x_{0}\right|+{||k||}_{L^{2}(0,T;V^{*})}\right\}. \end{array}$$

Hence if *k* is bounded in *L* ^2^(0,*T*;V*), then so is *x*_*k*_ in *𝒲*_1_(*T*) ≡ *L* ^2^(0,T;*V*)∩*W* ^1,2^(0,*T*;V*). Since *V* is compactly embedded in *H* by assumption, the embedding

(79) $$\begin{array}{c} {𝒲}_{1}\left(T\right)\subset L^{2}\left(0,T;H\right) \end{array}$$

is compact in view of Theorem 2 of Aubin [24]. Hence *k* ↦ *x*_*k*_ is compact from *L* ^2^(0,*T*;V*). Moreover, we have that *ℱ* is a compact mapping of

(80) $$\begin{array}{c} L^{2}\left(0,T;V^{*}\right)↪{𝒲}_{1}\left(T\right)↪L^{2}\left(0,T;H\right), \end{array}$$

which is of *L* ^2^(0,*T*;V*) to *L* ^2^(0,*T*;*H*).

## 5. Approximate Controllability

In this section, we show that the controllability of the corresponding linear equation is extended to the nonlinear differential equation. Let *U* be a Banach space of control variables. Here *C* is a linear bounded operator from *L* ^2^(0,*T*;*U*) to *L* ^2^(0,*T*;*H*), which is called a controller. For *x* ∈ *L* ^2^(0,*T*;*H*) we set

(81) $$\begin{array}{c} \left(Bx\right)\left(t\right)=\overset{t}{\underset{0}{\int }}N\left(t-s\right)x\left(s\right)ds, \end{array}$$

where *N* : [0,*∞*) → *ℒ*(*H*,*V*) is strongly continuous. Then it is immediately seen that *Bx* ∈ *C*([0,*T*];*V*) and hence *AS*(*s*)(*Bx*)(*s*) = *AS*(*s*)(*Bx*)(*s*) for 0 ≤ *s* ≤ *T* because *D*(*A*) = *V*. Since *t* → *N*(*t*) is strong continuous, by the uniform boundedness principle, there exists a constant *M*_*N*_ such that, for any *T* > 0,

(82) $$\begin{array}{c} \underset{t\in \left[0,T\right]}{\sup}{||AN\left(t\right)||}_{ℒ(H,V^{*})}\leq M_{N}. \end{array}$$

Consider the following neutral control equation

(83) ddt[x(t)+(Bx)(t)]=Ax(t)+f(t,x(t))+(Cu)(t),t∈(0,T],x(0)=x0,  (Bx)(0)=y0.

Let *x*(*T*;*B*,*f*,*u*) be a state value of the system (83) at time *T* corresponding to the operator *B*, the nonlinear term *f*, and the control *u*. We note that *S*(·) is the analytic semigroup generated by −*A*. Then the solution *x*(*t*;*B*,*f*,*u*) can be written as

(84) x(t;B,f,u) =S(t)(x0+y0)−(Bx)(t)  +∫0tS(t−s)    ×{A(Bx)(s)ds+f(s,x(s))+(Cu)(s)}ds.

And in view of Theorem 6,

(85) $$\begin{array}{c} {||x\left(\cdot ;B,f,u\right)||}_{{𝒲}_{1}(T)}\leq C_{3}\left(\left|x_{0}\right|+{||C||}_{ℒ(U,H)}{||u||}_{L^{2}(0,T;U)}\right). \end{array}$$

We define the reachable sets for the system (34) as follows:

(86) $$\begin{array}{c} R\left(T\right)=\left\{x\left(T;B,f,u\right):u\in L^{2}\left(0,T;U\right)\right\},\\ L\left(T\right)=\left\{x\left(T;0,0,u\right):u\in L^{2}\left(0,T;U\right)\right\}. \end{array}$$

**Definition 13**

The system (83) is said to be approximately controllable on [0,*T*] if for every *z*_*T*_ ∈ *H* and *ϵ* > 0 there exists a control function *u* ∈ *L* ^2^(0,*T*;*U*) such that the solution *x*(*T*;*B*,*f*,*u*) of (83) satisfies |*x*(*T*;*f*,*u*) − *z*_*T*_ | <*ϵ*; that is, $\bar{R_{T}(f)}=H$ , where $\bar{R(T)}$ is the closure of *R*(*T*) in *H*.

We define the linear operator $\hat{S}$ from *L* ^2^(0,*T*;*H*) to *H* by

(87) $$\begin{array}{c} \hat{S}p=\overset{T}{\underset{0}{\int }}S\left(T-s\right)p\left(s\right)ds \end{array}$$

for *p* ∈ *L* ^2^(0,*T*;*H*).

We need the following hypothesis.

**Assumption S**

(i) For any *ɛ* > 0 and *p* ∈ L^2^(0,*T*;*H*), there exists a *u* ∈ *L* ^2^(0,*T*;*U*) such that

(88)|S^p−S^Cu|<ɛ,(89)||Cu||L2(0,t;H)≤q1||p||L2(0,t;H), 0≤t≤T,

where *q*_1_ is a constant independent of *p*.

(ii) *f* is a nonlinear mapping of [0,*T*] × *H* into *H* satisfying the following.

There exists a function *L*_1_ : ℝ_+_ → ℝ such that

(90) $$\begin{array}{c} \left|f\left(t,x\right)-f\left(t,y\right)\right|\leq L_{1}\left(r\right)\left|x-y\right|,\;t\in \left[0,T\right], \end{array}$$

hold for |*x* | ≤*r* and |*y* | ≤*r*.

By virtue of condition (i) of Assumption S we note that *AS*(*t* − *s*)*Bx* = *S*(*t* − *s*)*A* *Bx* for each *x* ∈ *V*. Therefore, the system (83) is approximately controllable on [0,*T*] if for any *ɛ* > 0 and *z*_*T*_ ∈ *H* there exists a control *u* ∈ *L* ^2^(0,*T*;*U*) such that

(91) ||S(T)(x0+y0)−(Bx)(T) +S^{ABx+Fx+Cu}−zT||<ɛ,

where (*Fx*)(*t*) = *f*(*t*,*x*(*t*)) for *t* ≥ 0. Throughout this section, invoking (85), we can choose a constant *r*_1_ such that

(92) $$\begin{array}{c} r_{1}>C_{3}\left(\left|x_{0}\right|+{||C||}_{ℒ(U,H)}{||u||}_{L^{2}(0,T;U)}\right), \end{array}$$

and set

(93) $$\begin{array}{c} G\left(s,x\right)=A\left(Bx\right)\left(s\right)+f\left(s,x\left(s\right)\right). \end{array}$$

**Lemma 14**

Let *u*_1_ and *u*_2_ be in *L* ^2^(0,*T*;*U*). Then under the Assumption S, one has that, for 0 ≤ *t* ≤ *T*,

(94) |x(t;B,f,u1)−x(t;B,f,u2)| ≤MeM2t||Cu1−Cu2||L2(0,T;H),

where *M*_2_ = *e* ^*M*(*M*_*N*_ *T*+*L*_1_(*r*_1_))^.

**Proof**

Let *x*(*t*) = *x*(*t*;*B*,*f*,*u*_1_) and *x*_2_(*t*) = *x*(*t*;*B*,*f*,*u*_2_). Then for 0 ≤ *t* ≤ *T*, we have

(95) x1(t)−x2(t)=(Bx2)(t)−(Bx1)(t) +∫0tS(t−s){G(s,x1)−G(s,x2)}ds +∫0tS(t−s)C(u1(s)−u2(s))ds.

So we immediately obtain

(96) $$\begin{array}{c} \left|A\left(Bx_{2}\right)\left(t\right)-A\left(Bx_{1}\right)\left(t\right)\right|\leq M_{N}\overset{t}{\underset{0}{\int }}\left|x_{2}\left(s\right)-x_{1}\left(s\right)\right|ds, \end{array}$$

and so it holds that

(97) |∫0tS(t−s)A{(Bx2)(s)−(Bx1)(s)}ds|  ≤MMNT∫0t|x2(s)−x1(s)|ds.

Moreover, we have

(98) |∫0tS(t−s){f(s,x1(s))−f(s,x2(s))}ds|  ≤ML1(r1)∫0t|x2(s)−x1(s)|ds,|∫0tS(t−s){Cu1(s)−Cu2(s)}ds|  ≤Mt||Cu1−Cu2||L2(0,T1;V).

Thus, from (95) it follows that

(99) |x(t;B,f,u1)−x(t;B,f,u2)|  ≤Mt||Cu1−Cu2||L2(0,T;H)   +{MMNT+ML1(r1)}∫0t|x2(s)−x1(s)|ds.

Therefore, by using Gronwall's inequality this lemma follows.

**Theorem 15**

Under Assumption S, the system (83) is approximately controllable on [0,*T*].

**Proof**

We will show that $D(A)\subset \bar{R_{T}(g)}$ ; that is, for given *ɛ* > 0 and *z*_*T*_ ∈ *D*(*A*), there exists *u* ∈ *L* ^2^(0,*T*;*U*) such that

(100) $$\begin{array}{c} \left|z_{T}-x\left(T;B,f,u\right)\right|<ɛ, \end{array}$$

where

(101) x(T;B,f,u) =S(T)(x0+y0)−(Bx)(T)  +∫0TS(T−s){G(s,x(·;B,f,u))+Cu(s)}ds.

As *z*_*T*_ ∈ *D*(*A*) there exists *p* ∈ *L* ^2^(0,*T*;*Z*) such that

(102) $$\begin{array}{c} \hat{S}p=z_{T}+\left(Bx\right)\left(T\right)-S\left(T\right)\left(x_{0}+y_{0}\right); \end{array}$$

for instance, take *p*(*s*) = {(*z*_*T*_ + (*Bx*)(*T*)) − *sA*(*z*_*T*_ + (*Bx*)(*T*))} − *S*(*s*)(*x*_0_ + *y*_0_)/*T*. Let *u*_1_ ∈ *L* ^2^(0,*T*;*U*) be arbitrary fixed. Since by Assumption S there exists *u*_2_ ∈ *L* ^2^(0,*T*;*U*) such that

(103) $$\begin{array}{c} \left|\hat{S}\left(p-G\left(\cdot ,x\left(\cdot ,B,f,u_{1}\right)\right)\right)-\hat{S}Cu_{2}\right|<\frac{ɛ}{4}, \end{array}$$

it follows that

(104) |zT+(Bx)(T)−S(T)(x0+y0) − S^G(·,x(·B,f,u1))−S^Cu2|<ɛ4.

We can also choose *w*_2_ ∈ *L* ^2^(0,*T*;*U*) by Assumption S such that

(105) |S^(G(·x(·;B,f,u2))−G(·x(·;B,f,u1)))−S^Cw2|  <ɛ8

and by Assumption S

(106) ||Cw2||L2(0,t;H) ≤q1||G(·,x(·;B,f,u1))    − G(·,x(·;B,f,u2))||L2(0,t;H)

for 0 ≤ *t* ≤ *T*. Therefore, in view of Lemma 14 and Assumption S

(107) ||Cw2||L2(0,t;H) ≤q1{∫0t|G(τ,x(τ;B,f,u2))     − G(τ,x(τ;B,f,u1))|2dτ}1/2 ≤q1(MN+L(r1)){∫0t|x(τ;B,f,u2)          −x(τ;B,f,u1)|2dτ}1/2 ≤q1(MN+L(r1)){∫0t(MeM2)2          ×τ||Cu2−Cu1||L2(0,τ;H)2dτ}1/2 ≤q1(MN+L(r1))MeM2  ×(∫0tτdτ)1/2||Cu2−Cu1||L2(0,t;H) =q1(MN+L(r1))MeM2(t22)1/2||Cu2−Cu1||L2(0,t;H).

Put *u*_3_ = *u*_2_ − *w*_2_. We determine *w*_3_ such that

(108) |S^(G(·,x(·;B,f,u3))−G(·,x(·;B,f,u2))) −S^Cw3|<ɛ8,

(109) ||Cw3||L2(0,t;H) ≤q1||G(·,x(·;B,f,u3))    − G(·,x(·;B,f,u2))||L2(0,t;H)

for 0 ≤ *t* ≤ *T*. Hence, we have

(110) ||Cw3||L2(0,t;H) ≤q1{∫0t|G(τ,x(τ;B,f,u3))      − G(τ,x(τ;B,f,u2))|2dτ}1/2 ≤q1(MN+L(r1))  ×{∫0t|x(τ;B,f,u3)−x(τ;B,f,u2)|2dτ}1/2 ≤q1(MN+L(r1))MeM2  ×{∫0tτ||Cu3−Cu2||L2(0,τ:H)2dτ}1/2 ≤q1(MN+L(r1))MeM2  ×{∫0tτ||Cw2||L2(0,τ;H)2dτ}1/2 ≤q1(MN+L(r1))MeM2  ×{∫0tτ(q1(MN+L(r1))MeM2)2τ22    × ||Cu2−Cu1||L2(0,τ;H)2dτ}1/2 ≤(q1(MN+L(r1))MeM2)2  ×(∫0tτ32dτ)1/2||Cu2−Cu1||L2(0,t;H) =(q1(MN+L(r1))MeM2)2  ×(t42·4)1/2||Cu2−Cu1||L2(0,t;H).

By proceeding with this process and from

(111) ||C(un−un+1)||L2(0,t;H) =||Cwn||L2(0,t;H)≤(q1(MN+L(r1))MeM2)n−1  ×(t2n−22·4⋯(2n−2))1/2||Cu2−Cu1||L2(0,t;H) =(q1(MN+L(r1))MeM2t2)n−1  ×1(n−1)!||Cu2−Cu1||L2(0,t;H),

it follows that

(112) ∑n=1∞||Cun+1−Cun||L2(0,T;H) ≤∑n=0∞(q1T(MN+L(r1))MeM22)n  ×1n!||Cu2−Cu1||L2(0,T;H)<∞.

Therefore, there exists u* ∈ *L* ^2^(0,*T*;*H*) such that

(113) $$\begin{array}{c} \underset{n\to \infty }{\lim}Cu_{n}=u^{*}\;\text{in}\;L^{2}\left(0,T;H\right). \end{array}$$

From (104), (105) it follows that

(114) |zT+(Bx)(T)−S(T)(x0+y0)  − S^G(·,x(·;B,f,u2))−S^Cu3| =|zT+(Bx)(T)−S(T)(x0+y0)   −S^G(·,x(·;B,f,u1))−S^Cu2+S^Cw2   −S^[G(·,x(·;B,f,u2))−G(·,x(·;B,f,u1))]| <(122+123)ɛ.

By choosing *w*_*n*_ ∈ *L* ^2^(0,*T*;*U*) by Assumption B, such that

(115) |S^(G(·x(·;B,f,un))−G(·x(·;B,f,un−1)))−S^Cwn| <ɛ2n+1,

putting *u*_*n*+1_ = *u*_*n*_ − *w*_*n*_, we have

(116) |zT+(Bx)(T)−S(T)(x0+y0) − S^G(·,x(·;B,f,un))−S^Cun+1| <(122+⋯+12n+1)ɛ, n=1, 2,….

Therefore, for *ɛ* > 0 there exists integer *N* such that

(117) |S^CuN+1−S^CuN|<ɛ2,|zT+(Bx)(T)−S(T)(x0+y0) − S^G(·,x(·;B,f,uN))−S^CuN| ≤|zT+(Bx)(T)−S(T)(x0+y0)   − S^G(·,x(·;B,f,uN))−S^CuN+1|  +|S^CuN+1−S^CuN| <(122+⋯+12N+1)ɛ+ɛ2≤ɛ.

Thus the system (83) is approximately controllable on [0,*T*] as *N* tends to infinity.

**Example 16**

Let

(118) $$\begin{array}{c} H=L^{2}\left(0,\pi \right),\;\;V=H_{0}^{1}\left(0,\pi \right),\;\;V^{*}=H^{-1}\left(0,\pi \right),\\ a\left(u,v\right)=\overset{\pi }{\underset{0}{\int }}\frac{du\left(y\right)}{dy}\;\frac{\bar{dv\left(y\right)}}{dy}dy,\\ A=\frac{\partial^{2}}{\partial y^{2}}\;\text{with}\;\;D\left(A\right)=\left\{x\in H^{2}\left(0,\pi \right):x\left(0\right)=x\left(\pi \right)=0\right\}. \end{array}$$

The eigenvalue and the eigenfunction of *A* are *λ*_*n*_ = −*n* ^2^ and *ϕ*_*n*_(*y*) = (2/*π*)^1/2^sin*ny*, respectively. Moreover,

(a) {*ϕ*_*n*_ : *n* ∈ *N*} is an orthogonal basis of *H*,
(b) *S*(*t*)*x* = ∑_*n*=1_^*∞*^ *e* ^*n* ^2^ *t*^(*x*, *ϕ*_*n*_)*ϕ*_*n*_,  ∀*x* ∈ *H*, *t* > 0,
(c) let 0 < *α* < 1; then the fractional power *A* ^*α*^ : *D*(*A* ^*α*^) ⊂ *H* → *H* of *A* is given by
(119) $$\begin{array}{c} A^{\alpha }x=\overset{\infty }{\underset{n=1}{\sum }}n^{2\alpha }\left(x,\phi_{n}\right)\phi_{n},\;\;D\left(A^{\alpha }\right):=\left\{x:A^{\alpha }x\in H\right\}. \end{array}$$

In particular, *A* ^−1/2^ *x* = ∑_*n*=1_^*∞*^(1/*n*)(*x*,*ϕ*_*n*_)*ϕ*_*n*_ and ||*A* ^−1/2^|| = 1.

Consider the following neutral differential control system:

(120) ∂∂t[x(t,y)+∫0t∫0πb(t−s,z,y)x(s,z)dz ds]=Ax(t,y)+g′(|x(t,y)|2)x(t,y)+(Cu)(t),               t∈(0,T],x(t,0)=x(t,π0)=0,

where *g* is a real valued function belonging to *C* ^2^([0,*∞*)) which satisfies the following conditions:

(i) *g*(0) = 0, *g*(*r*) ≥ 0 for *r* > 0,
(ii) *g*′(*r*) ≤ *c*(*r* + 1) and |*g*′′(*r*)|≤*c* for *r* ≥ 0 and *c* > 0. If we present
(121) $$\begin{array}{c} f\left(x\left(t,y\right)\right)=g^{\prime }\left({\left|x\left(t,y\right)\right|}^{2}\right)x\left(t,y\right), \end{array}$$
  *f* is a mapping from the whole *V* to *H* by Sobolev's imbedding theorem (see [21], Theorem 6.1.6). As an example of *g* in the above, we can choose *g*(*r*) = *μ* ^2^ *r* + *η* ^2^ *r* ^2^/2 (*μ* and *η* are constants). In addition, we need to impose the following conditions (see [7, 25]).
(iii) The function *b* is measurable and
(122) ∫0π∫0t∫0πb2(t−s,z,y)dz ds dy<∞.
(iv) The function (∂^2^/∂*z* ^2^)*b* is measurable, *b*(0,*y*,*π*) = *b*(0,*y*,0), and
(123) $$\begin{array}{c} M_{b}:=\overset{\pi }{\underset{0}{\int }}\overset{t}{\underset{0}{\int }}\overset{\pi }{\underset{0}{\int }}{\left(\frac{\partial }{\partial z}b\left(t-s,z,y\right)\right)}^{2}dz\;ds\;dy<\infty . \end{array}$$
(v) *C* : *L* ^2^(0,*T*;*U*) → *L* ^2^(0,*T*;*H*) is a bounded linear operator.

We define *B* : *L* ^2^(0,*T*;*V*) → *L* ^2^(0,*T*;*H*) by

(124) $$\begin{array}{c} \left(Bx\right)\left(t\right)=\overset{t}{\underset{0}{\int }}\overset{\pi }{\underset{0}{\int }}b\left(t-s,z,y\right)x\left(s,z\right)dy\;ds. \end{array}$$

From (ii) it follows that *B* is bounded linear and

(125) A1/2(Bx)(t) =1n2π((Bx)(t),sin⁡ny)ϕn =2π(∫0t∫0π∂∂yb(t−s,z,y)dy ds,cos⁡⁡ny)ϕn =2π((B1x)(t),cos⁡⁡ny)ϕn,

where

(126) $$\begin{array}{c} \left(B_{1}x\right)\left(t\right)=\overset{t}{\underset{0}{\int }}\overset{\pi }{\underset{0}{\int }}\frac{\partial }{\partial y}b\left(t-s,z,y\right)dyds. \end{array}$$

Thus, by (iv) the operator *B*_1_ is bounded linear with $||B_{1}||\leq \sqrt{M_{b}}$ and we have that *B* ∈ *D*(*A* ^1/2^) and ||*A* ^1/2^ *Bx*|| = ||*B*_1_ *x*||. Therefore from Theorem 6, there exists a solution *x* of (120) such that

(127) $$\begin{array}{c} x\in L^{2}\left(0,T;V\right)\cap W^{1,2}\left(0,T;V^{*}\right)\subset C\left(\left[0,T\right];H\right). \end{array}$$

Moreover, from Theorem 15 the neutral system (120) is approximately controllable on [0, *T*].
